# Supplementary material for: Integrated ATAC-Seq and RNA-Seq Data Analysis to Reveal OsbZIP14 Function in Rice in Response to Heat Stress
Source: Int J Mol Sci. 2023 Mar 15;24(6):5619. doi: 10.3390/ijms24065619 (PMC10057503; doi:10.3390/ijms24065619)
Supplement: Supplementary file 1 [file ijms-24-05619-s001.zip › ijms-2277975-supplementary.pdf]

Table S1 Data of tested rice under high temperature stress downloaded from this study

NCBI login number	Rice varieties tested	Sample	Sequencing type
PRJNA604026	Japonica rice (Oryza sativa L.) cultivar Nipponbare	leaf	RNA-seq
PRJNA604028	Japonica rice (Oryza sativa L.) cultivar Nipponbare	leaf	ATAC-seq

Table S2 Rice genes with heat stress motifs mapped against known Arabidopsis Transcription factors

Subject name	Query name	Identity(%)	E-value	Score
AT3G02990.1	Os03g0161900	95.122	2.10E-09	65.8
AT1G22640.1	Os02g0642050	90.909	6.24E-08	60.2
AT1G22640.1	Os02g0641900	88	6.24E-08	60.2
AT5G16820.1	Os03g0795900	76.423	2.61E-28	128
AT5G16820.1	Os07g0178600	76.695	9.38E-28	126
AT5G16820.1	Os01g0749300	76.151	5.65E-25	117
AT5G16820.1	Os10g0419300	75	2.03E-24	115
AT5G16820.1	Os07g0640900	79.286	3.42E-17	91.6
AT5G16820.1	Os03g0366800	81.25	1.23E-16	89.8
AT5G16820.1	Os02g0527300	82	5.73E-15	84.2
AT5G16820.1	Os09g0526650	82.716	4.46E-11	71.3
AT5G16820.1	Os09g0526600	79.245	4.46E-11	71.3
AT5G16820.1	Os08g0546800	93.333	5.77E-10	67.6
AT5G16820.1	Os06g0553100	87.755	3.47E-07	58.4
AT2G18160.1	Os02g0132500	80.435	2.86E-11	71.3
AT3G06490.1	Os11g0684000	79.592	1.18E-31	139
AT3G06490.1	Os01g0128000	73.913	4.30E-21	104
AT3G06490.1	Os12g0564100	83.019	7.20E-19	97.1
AT3G06490.1	Os04g0508500	95.349	1.57E-10	69.4
AT3G06490.1	Os03g0142600	95.349	1.57E-10	69.4

AT3G06490.1	Os02g0618400	79.208	1.57E-10	69.4
AT3G06490.1	Os09g0538400	95	7.30E-09	63.9
AT3G06490.1	Os02g0618525	100	7.30E-09	63.9
AT3G06490.1	Os01g0637800	77.778	9.45E-08	60.2
AT3G06490.1	Os12g0175400	94.595	3.40E-07	58.4
AT3G06490.1	Os11g0181000	90.698	3.40E-07	58.4
AT3G06490.1	Os11g0180900	94.595	3.40E-07	58.4
AT3G06490.1	Os08g0549000	94.595	3.40E-07	58.4
AT3G06490.1	Os05g0429900	94.595	3.40E-07	58.4
AT3G06490.1	Os03g0578900	94.595	3.40E-07	58.4
AT3G06490.1	Os03g0303500	94.595	3.40E-07	58.4
AT3G06490.1	Os01g0874300	94.595	3.40E-07	58.4
AT3G06490.1	Os01g0169000	94.595	3.40E-07	58.4
AT3G59350.1	Os01g0323100	78.74	1.67E-65	252
AT1G79180.1	Os02g0624350	94.737	6.89E-08	60.2
AT1G79180.1	Os02g0624300	85.714	6.89E-08	60.2
AT5G66730.1	Os02g0518500	73.597	7.76E-45	183

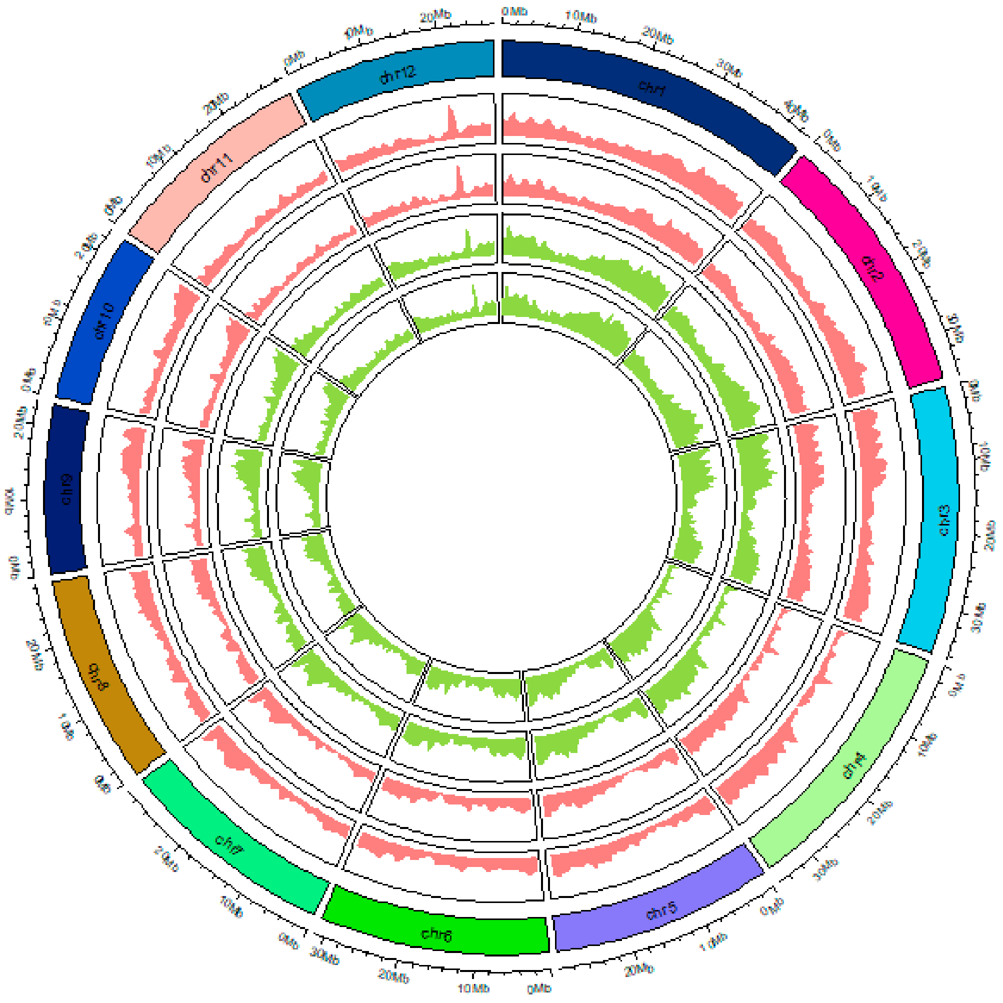

Figure S1. Circos plots of ATAC-seq sequencing fragments of all chromosomes in rice

(red and green peaks represent two biological replicate sequenced fragments from the 36h blank and experimental groups, respectively, and the outermost regions of different colors represent different chromosomes of rice.)

Table S3 Differential THSs identified after heat treatment

positively enriched THSs	negatively enriched THSs	Percentage of diffential THSs
1692	156	7.07%

A

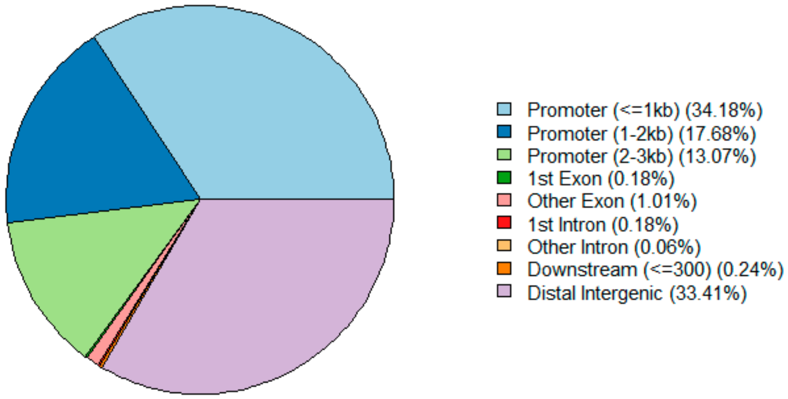

B

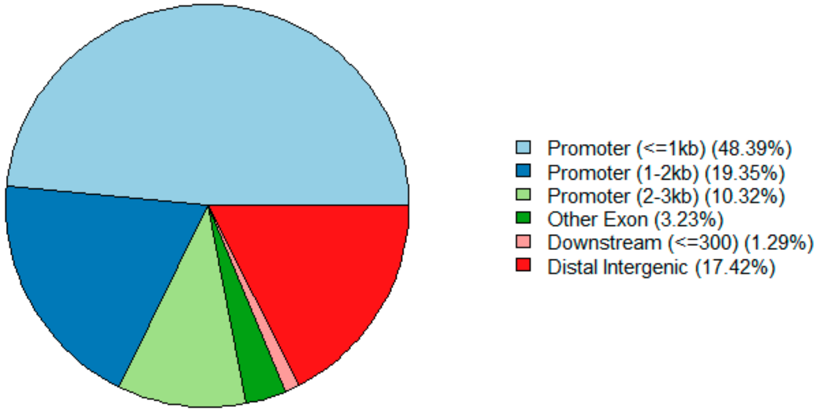

Figure S2. Quantity and Genome Distribution of Differentially Enriched THS after Heat Treatment

(A is the proportion of differentially enriched THSs, B is the positive distribution of THSs in the genome after high temperature treatment, and c is the negative distribution of the THSs after high temperature treatment.)

Total Target Sequences = 1692, Total Background Sequences = 43169

Rank	Motif	Name	P-value	log P-pvalue	q-value (Benjamini)	# Target Sequences with Motif	% of Targets Sequences with Motif	# Background Sequences with Motif	% of Background Sequences with Motif
1	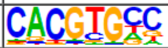	IBL1(bHLH)/Seedling-IBL1-ChIP-Seq(GSE51120)/Homer	1e-60	-1.395e+02	0.0000	936.0	55.32%	15368.3	35.60%
2	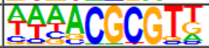	CAMTA1(CAMTA)/col-CAMTA1-DAP-Seq(GSE60143)/Homer	1e-54	-1.251e+02	0.0000	549.0	32.45%	7286.6	16.88%
3	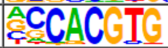	c-Myc(bHLH)/LNCAP-cMyc-ChIP-Seq(Unpublished)/Homer	1e-47	-1.091e+02	0.0000	430.0	25.41%	5348.5	12.39%
4	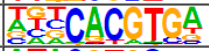	SPCH(bHLH)/Seedling-SPCH-ChIP-Seq(GSE57497)/Homer	1e-40	-9.273e+01	0.0000	507.0	29.96%	7245.8	16.79%
5	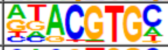	HIF-1b(HLH)/T47D-HIF1b-ChIP-Seq(GSE59937)/Homer	1e-39	-9.084e+01	0.0000	720.0	42.55%	11887.1	27.54%
6	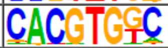	ABF1(bZIP)/Arabidopsis-ABF1-ChIP-Seq(GSE80564)/Homer	1e-37	-8.597e+01	0.0000	464.0	27.42%	6558.9	15.19%
7	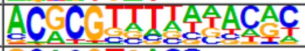	CAMTA5(CAMTA)/col-CAMTA5-DAP-Seq(GSE60143)/Homer	1e-32	-7.382e+01	0.0000	264.0	15.60%	3066.7	7.10%
8	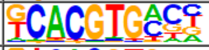	bHLHE41(bHLH)/proB-Bhlhe41-ChIP-Seq(GSE93764)/Homer	1e-31	-7.339e+01	0.0000	583.0	34.46%	9439.5	21.87%
9	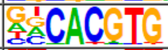	BMAL1(bHLH)/Liver-Bmal1-ChIP-Seq(GSE39860)/Homer	1e-28	-6.494e+01	0.0000	576.0	34.04%	9587.1	22.21%
10	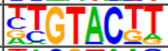	SPL9(SBP)/colamp-SPL9-DAP-Seq(GSE60143)/Homer	1e-27	-6.436e+01	0.0000	741.0	43.79%	13369.5	30.97%
11	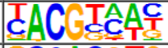	ATAF1(NAC)/col-ATAF1-DAP-Seq(GSE60143)/Homer	1e-27	-6.356e+01	0.0000	1040.0	61.47%	20779.2	48.14%
12	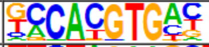	NPAS2(bHLH)/Liver-NPAS2-ChIP-Seq(GSE39860)/Homer	1e-27	-6.230e+01	0.0000	366.0	21.63%	5257.4	12.18%
13	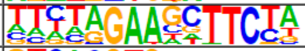	HSF6(HSF)/col-HSF6-DAP-Seq(GSE60143)/Homer	1e-26	-6.196e+01	0.0000	64.0	3.78%	288.9	0.67%
14	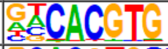	CLOCK(bHLH)/Liver-Clock-ChIP-Seq(GSE39860)/Homer	1e-25	-5.924e+01	0.0000	267.0	15.78%	3435.8	7.96%
15	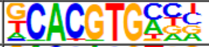	bHLHE40(bHLH)/HepG2-BHLHE40-ChIP-Seq(GSE31477)/Homer	1e-25	-5.897e+01	0.0000	183.0	10.82%	1964.8	4.55%
16	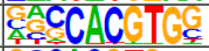	n-Myc(bHLH)/mES-nMyc-ChIP-Seq(GSE11431)/Homer	1e-24	-5.590e+01	0.0000	325.0	19.21%	4630.9	10.73%
17	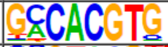	E-box/Arabidopsis-Promoters/Homer	1e-23	-5.506e+01	0.0000	314.0	18.56%	4438.7	10.28%
18	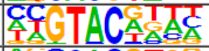	SPL1(SBP)/colamp-SPL1-DAP-Seq(GSE60143)/Homer	1e-23	-5.503e+01	0.0000	645.0	38.12%	11540.7	26.74%
19	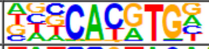	PIF4(bHLH)/Seedling-PIF4-ChIP-Seq(GSE35315)/Homer	1e-23	-5.415e+01	0.0000	396.0	23.40%	6118.0	14.17%
20	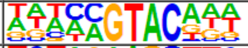	SPL15(SBP)/colamp-SPL15-DAP-Seq(GSE60143)/Homer	1e-23	-5.358e+01	0.0000	563.0	33.27%	9742.0	22.57%
21	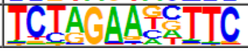	AT2G01818(PLATZ)/col-AT2G01818-DAP-Seq(GSE60143)/Homer	1e-23	-5.300e+01	0.0000	53.0	3.13%	231.5	0.54%

Figure S3. Positive enriched motif identified by ATAC-seq (partial)

Rank	Motif	Name	P-value	log P-pvalue	q-value (Benjamini)	# Target Sequences with Motif	% of Targets Sequences with Motif	# Background Sequences with Motif	% of Background Sequences with Motif
1	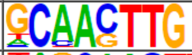	bHLH130(bHLH)/col-bHLH130-DAP-Seq(GSE60143)/Homer	1e-5	-1.162e+01	0.0091	28.0	17.95%	3580.0	7.31%
2	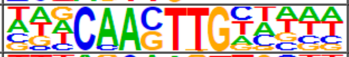	bHLH122(bHLH)/col100-bHLH122-DAP-Seq(GSE60143)/Homer	1e-3	-8.227e+00	0.1344	27.0	17.31%	4124.1	8.42%
3	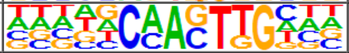	bHLH80(bHLH)/col-bHLH80-DAP-Seq(GSE60143)/Homer	1e-3	-7.314e+00	0.2235	27.0	17.31%	4374.4	8.93%
4	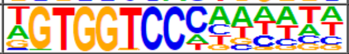	TCP3(TCP)/colamp-TCP3-DAP-Seq(GSE60143)/Homer	1e-3	-7.123e+00	0.2235	11.0	7.05%	1090.8	2.23%
5	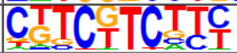	Unknown4/Arabidopsis-Promoters/Homer	1e-2	-6.630e+00	0.2657	36.0	23.08%	6796.6	13.88%
6	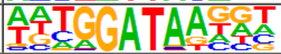	AT4G00250(GeBP)/col-AT4G00250-DAP-Seq(GSE60143)/Homer	1e-2	-6.459e+00	0.2657	24.0	15.38%	3927.5	8.02%
7	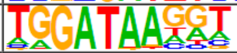	AT5G56840(MYBrelated)/colamp-AT5G56840-DAP-Seq(GSE60143)/Homer	1e-2	-6.002e+00	0.3555	39.0	25.00%	7827.1	15.98%
8	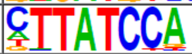	At5g47390(MYBrelated)/col-At5g47390-DAP-Seq(GSE60143)/Homer	1e-2	-5.908e+00	0.3555	39.0	25.00%	7869.0	16.07%
9	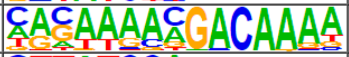	At1g14580(C2H2)/colamp-At1g14580-DAP-Seq(GSE60143)/Homer	1e-2	-5.527e+00	0.4448	9.0	5.77%	964.2	1.97%
10	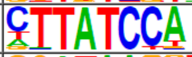	AT5G61620(MYBrelated)/colamp-AT5G61620-DAP-Seq(GSE60143)/Homer	1e-2	-5.482e+00	0.4448	42.0	26.92%	8865.4	18.10%
11	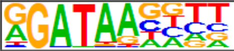	At1g49010(MYBrelated)/col-At1g49010-DAP-Seq(GSE60143)/Homer	1e-2	-5.424e+00	0.4448	49.0	31.41%	10813.3	22.08%
12	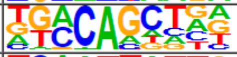	bZIP18(bZIP)/colamp-bZIP18-DAP-Seq(GSE60143)/Homer	1e-2	-5.173e+00	0.4753	82.0	52.56%	20652.2	42.17%
13	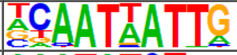	HAT5(Homeobox)/colamp-HAT5-DAP-Seq(GSE60143)/Homer	1e-2	-4.927e+00	0.5608	9.0	5.77%	1060.2	2.16%
14	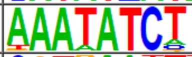	At4g01280(MYBrelated)/colamp-At4g01280-DAP-Seq(GSE60143)/Homer	1e-2	-4.834e+00	0.5717	20.0	12.82%	3494.2	7.13%
15	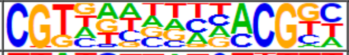	ANAC042(NAC)/col-ANAC042-DAP-Seq(GSE60143)/Homer	1e-2	-4.802e+00	0.5717	24.0	15.38%	4481.2	9.15%
16	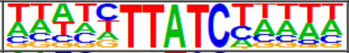	At5g58900(MYBrelated)/colamp-At5g58900-DAP-Seq(GSE60143)/Homer	1e-2	-4.781e+00	0.5717	42.0	26.92%	9222.8	18.83%
17	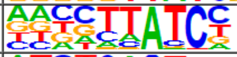	At5g08520(MYBrelated)/colamp-At5g08520-DAP-Seq(GSE60143)/Homer	1e-2	-4.751e+00	0.5717	30.0	19.23%	6027.9	12.31%
18	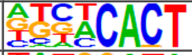	AZF1(C2H2)/colamp-AZF1-DAP-Seq(GSE60143)/Homer	1e-2	-4.716e+00	0.5717	85.0	54.49%	21897.3	44.71%
19	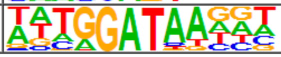	At1g19000(MYBrelated)/colamp-At1g19000-DAP-Seq(GSE60143)/Homer	1e-2	-4.633e+00	0.5717	29.0	18.59%	5820.0	11.88%

Figure S4. The negatively enriched motif identified by ATAC-seq

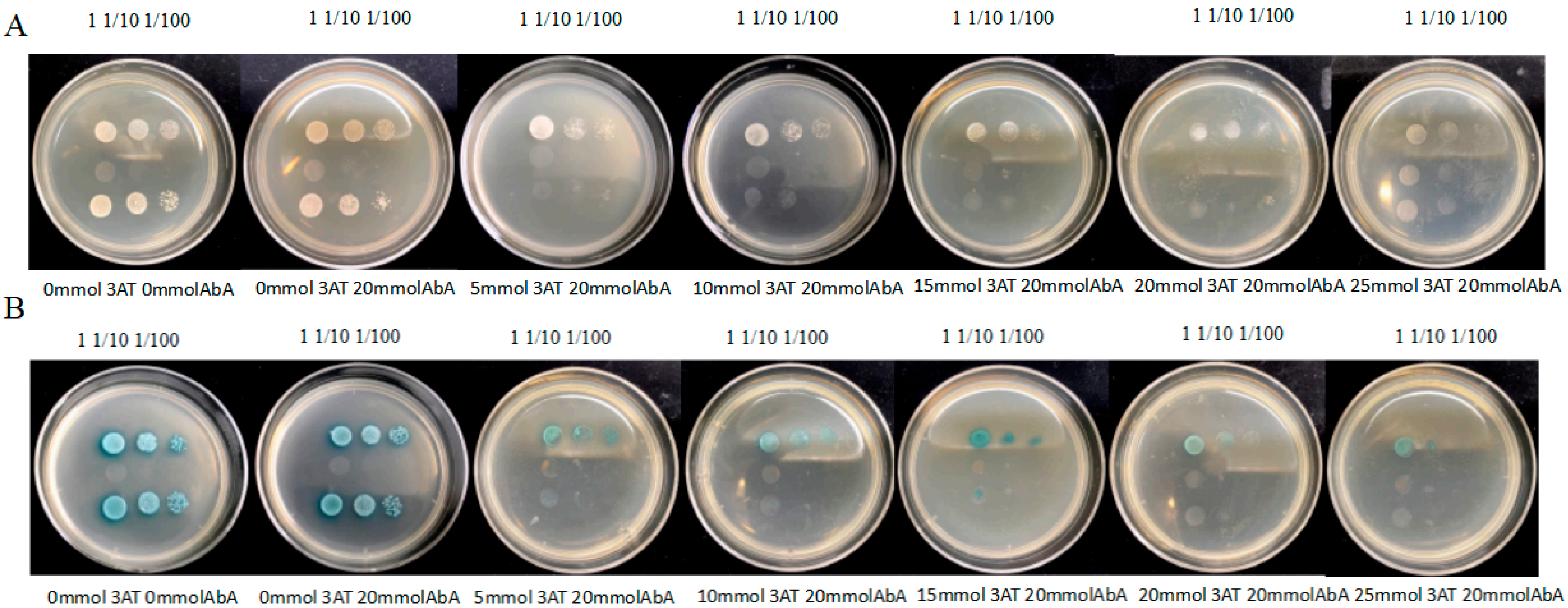

Figure S5. *OsbZIP14* Self activation Activity Inhibition in Three and Four Deficiency Plates

A

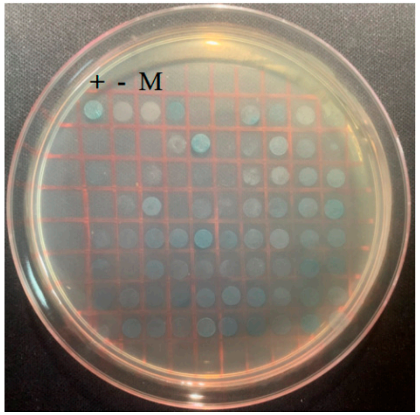

B

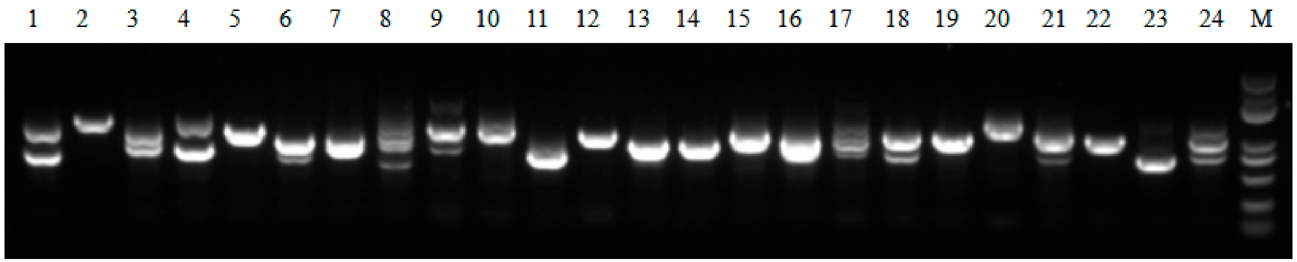

C

	NCBI accession	Gene annotation	Sequence similarity	E-value
1	XM_026021003.1	CCR4-NOT transcription complex subunit 1	97.56%	0
2	XM_015780209.2	photosystem I assembly protein	99.43%	0
3	AK072521.1	cDNA clone:J023131B06	98.49%	0
4	XM_015785159.2	uncharacterized	98.70%	0
5	KC140124.1	NLS receptor mRNA	97.94%	0
6	XM_015787757.2	uncharacterized	99.42%	0
7	XM_015789930.2	bZIP transcription factor	100.00%	0
8	XM_015789238.2	protein PELPK1	99.81%	0

Figure S6. Library screening of *OsbZIP14* interaction protein. A is the yeast monoclone grown on the solid medium containing 20mmol AbA and 3-AT containing X-α-Gal,+ is the positive control, and - is the negative control; B is the PCR detection result of inserted fragments in partial cDNA library, and M is Maker=5000bp. Some Potential Interacting Proteins of *OsbZIP14* in C.

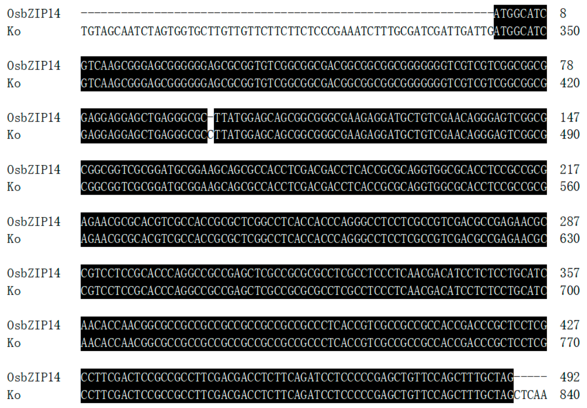

Figure S7. *OsbZIP14* Transgenic knockout type
